# Discriminability effect on Garner interference: evidence from recognition of facial identity and expression

**DOI:** 10.3389/fpsyg.2013.00943

**Published:** 2013-12-19

**Authors:** Yamin Wang, Xiaolan Fu, Robert A. Johnston, Zheng Yan

**Affiliations:** ^1^Beijing Key Laboratory of Learning and Cognition, Department of Psychology, Capital Normal UniversityBeijing, China; ^2^State Key Laboratory of Brain and Cognitive Science, Institute of Psychology, Chinese Academy of SciencesBeijing, China; ^3^School of Psychology, Keynes College, University of KentCanterbury, UK; ^4^University at Albany, State University of New YorkAlbany, NY, USA

**Keywords:** discriminability, Garner interference, facial recognition, facial identity recognition, facial expression recognition

## Abstract

Using Garner’s speeded classification task existing studies demonstrated an asymmetric interference in the recognition of facial identity and facial expression. It seems that expression is hard to interfere with identity recognition. However, discriminability of identity and expression, a potential confounding variable, had not been carefully examined in existing studies. In current work, we manipulated discriminability of identity and expression by matching facial shape (long or round) in identity and matching mouth (opened or closed) in facial expression. Garner interference was found either from identity to expression (Experiment 1) or from expression to identity (Experiment 2). Interference was also found in both directions (Experiment 3) or in neither direction (Experiment 4). The results support that Garner interference tends to occur under condition of low discriminability of relevant dimension regardless of facial property. Our findings indicate that Garner interference is not necessarily related to interdependent processing in recognition of facial identity and expression. The findings also suggest that discriminability as a mediating factor should be carefully controlled in future research.

## GARNER INTERFERENCE IN RECOGNITION OF FACIAL IDENTITY AND EXPRESSION

To understand the mechanisms underlying face recognition, Bruce and Young (1986) presented the functional model of face recognition, in which facial properties, such as facial identity and facial expression, are processed separately. A large number of studies have provided evidence for the independent relationship (e.g., Tranel et al., 1988; Young et al., 1993; Campbell et al., 1996; see Calder and Young, 2005 for a review). But there are some other studies that support a connection between them (e.g., Hasselmo et al., 1989; Sugase et al., 1999; Goshen-Gottstein and Ganel, 2000; Ganel et al., 2005). Current work focuses on the studies of Garner interference between facial identity and facial expression. By demonstrating Garner interference, existing studies tend to support interdependent processing of identity and expression (e.g., Schweinberger and Soukup, 1998; Schweinberger et al., 1999; Ganel and Goshen-Gottstein, 2004). But the role of discriminability of identity and expression has not been carefully examined.

Garner interference is typically measured with a Garner’s speeded-classification task, in which participants are instructed to direct selective attention to a specific dimension of an object (Garner, 1974) or a face (Schweinberger and Soukup, 1998) while ignoring its other irrelevant dimensions. For a given pair of dimensions belonging to a single object or a face, Garner’s task can be used to measure whether one dimension (e.g., identity) can be processed without being influenced by the other, irrelevant dimension (e.g., expression) and, similarly, whether the other dimension (e.g., expression) can be processed without being influenced by the irrelevant first dimension (e.g., identity). If the processing of each of the dimensions, when it is defined to be the relevant dimension, is not influenced by the other, irrelevant dimension, then the two dimensions are labeled *separable dimensions*. If, however, the processing of each of the dimensions cannot be made without interference from the other, irrelevant dimension, the two dimensions were labeled *integral dimensions* (Ganel and Goshen-Gottstein, 2004).

In facial recognition field, Garner’s task has recently been used as a tool to test the relationship between facial identity and expression. In the first example, Schweinberger and his collaborators conducted a series of experiments using Garner’s classic speeded classification task (e.g., Schweinberger and Soukup, 1998; Schweinberger et al., 1999). In these studies, interference from identity to expression recognition was consistently observed, but interference from expression to identity was scarcely found (Schweinberger and Soukup, 1998; Schweinberger et al., 1999), especially in unfamiliar face recognition (Kaufmann and Schweinberger, 2004), thus leading to the asymmetric relations hypothesis, which states identity is perceived independently of, but may exert an influence on expression. In the second example, Ganel and Goshen-Gottstein (2004) conducted a series of experiments in which interference was found not only from identity to expression, but also from expression to identity. Based on their findings, these researchers advanced the *structural-reference* hypothesis, suggesting that the systems of processing identity and expression are closely interconnected rather than completely separate, wherein the structure of the face serves as an existing reference to help process expression information efficiently, while unique expression also serves as an existing reference to help process the identity of a face (Ganel and Goshen-Gottstein, 2004).

Both the Schweinberger’s group and the Ganel’s group initiated new and productive lines of experimental research and demonstrated alternative models of the processing of facial identity and expression recognition. Thus, together with the research supporting the interdependent model, their studies have contributed to further understanding the richness and complexity of the facial recognition processes. More importantly, their studies not only added new knowledge to the existing face recognition literature but also pointed to important directions for future research. Among these new directions is understanding the role of discriminability on the processing of facial identity and expression recognition.

In studies by both research groups, the effects of familiarity as one central mediating variable on processing identity and expression have been carefully excluded (e.g., Schweinberger and Soukup, 1998) or systematically examined (e.g., Ganel and Goshen-Gottstein, 2004), suggesting that whether one sees familiar or unfamiliar faces influences facial recognition, and that familiar and unfamiliar facial recognition might have different underlying mechanisms (Young et al., 1986; Rossion et al., 2001). In contrast, effects of discriminability as a variable on recognition of facial identity and expression have been either controlled (e.g., Ganel and Goshen-Gottstein, 2004) or only partially studied (e.g., Schweinberger and Soukup, 1998) but not fully manipulated to examine the effects of discriminability.

## EFFECT OF DISCRIMINABILITY ON GARNER INTERFERENCE IN FACIAL RECOGNITION

Recently, Graham and LaBar (2007) again observed the effect of discriminability on recognition of facial expression and gaze. By increasing the discriminability of gaze, they demonstrated interference to each other instead of asymmetric interference. Beyond facial recognition, several previous studies in Garner interference demonstrated the effect of discriminability (Melara and Marks, 1990; Melara and Mounts, 1993; Algom et al., 1996; Sabri et al., 2001). Based on existing researches, we propose that Garner interference in facial recognition does not directly reflect an interdependent relationship between these facial properties. It is a type of general interference in facial recognition, in which discriminability of facial cue plays a part in causing this interference. We call this the *mediating discriminability hypothesis*. In terms of this proposed hypothesis, we can expect that systematically manipulated discriminability of both facial identity and facial expression would predict significant or insignificant Garner interference. To grasp a further understanding of facial recognition, it is necessary to examine this discriminability effect.

In facial recognition, discriminability describes the difficulty of recognizing or differentiating facial cues. For example, the discriminability of expression indicates the difficulty of determining the difference among expressions. By this definition, discriminability is a relative variable. The term high or low discriminability, used in the current work, refers to discriminability that is relatively high or low, except when otherwise stated.

To fully examine the effect of discriminability on the perception of facial identity and facial expression, the variable, discriminability, should be placed on the front stage. It should be systematically manipulated in the following 2 × 2 combinations (high and low discriminability; facial identity, and facial expression): high discriminability in identity with low discriminability in expression; low discriminability in identity with high discriminability in expression; both low discriminability in identity and expression; and both high discriminability in identity and expression. Among these combinations, low discriminability in identity with high discriminability in expression and both high discriminability get no examination in the existing studies.

A previous opinion proposed that a more discriminated irrelevant dimension might interfere with relevant dimension in Garner task. We argue that it is not irrelevant dimension but less discriminated relevant dimension causes interference from irrelevant dimension. Relevant dimension with low discriminability leads to a reference to irrelevant dimension in facial discrimination task. Such a reference to other facial information in a hard recognition task exactly demonstrates the flexibility of our recognition. The existing evidence suggests that even if the discriminability of facial identity is as low as expression, reference to identity was found in facial expression recognition (Ganel and Goshen-Gottstein, 2004).

According to the above analysis on discriminability, we propose that discriminability within facial cue, but not between facial cues, are responsible for Garner effect in recognition of facial identity and expression. In terms of the mediating discriminability hypothesis, low levels of discriminability in relevant dimension causes interference from irrelevant dimension.

This hypothesis makes four predictions: (1) if discriminability of expression is low but discriminability of identity is high, then interference from identity to expression would be found; (2) if discriminability of identity is low but discriminability of expression is high, then interference from expression to identity would be found; (3) if discriminability of expression and identity are both low, the interference both from identity to expression and from expression to identity would be observed; and (4) if discriminability of expression and identity are both high, then no interference would be observed.

To test the validity of the mediating discriminability hypothesis, we designed four similar experiments (labeled as Experiment 1–4) where each of them was intended to validate one of the four predictions of the hypothesis. We systematically manipulated the variable of discriminability for identity and expression by exhausting four possible combinations: low expression discriminability and high identity discriminability, low identity discriminability and high expression discriminability, low expression and identity discriminability, and high expression and identity discriminability. With these selected four groups of face photos, we measured the Garner interference in Experiments 1–4, as did the Schweinberger group and the Ganel group. To best assess the effects of discriminability, we restricted our research to unfamiliar face recognition.

## EXPERIMENT 1

The purpose of this experiment was to test the first prediction of the mediating discriminability hypothesis: if discriminability of facial expression is low and discriminability of facial identity is high, then identity will interfere with expression but expression will not influence identity recognition.

### METHOD

#### Participants

Forty-eight undergraduates with normal or corrected-to-normal vision were recruited in the experiment. Half contributed to the discriminability assessment and half contributed to the Garner effect measurements. Among the participants for discriminability assessment, 12 participated in the identity discriminability assessment and the other 12 completed the expression discriminability assessment. Similarly, participants for Garner effect measurements were also randomly assigned to identity judgment task and expression judgment task. As a result, 12 participants attended discriminability assessment before the other 12 participants took part in Garner effect measurements in either identity or expression recognition. To the end, we used the results of discriminability assessments to quantitatively show the discriminability levels of selected facial photos, and used the results from Garner effect measurements to test the first prediction.

#### Stimuli

In terms of our hypothesis, we needed to manipulate discriminability of facial identity and expression. Face photos used in all our experiments must be manipulated to meet the requirements of discriminability combinations.

The goal to define and manipulate discriminability with a perfect quantitative index is challenging. Discriminability might be a function of a range of factors. The scale like selective response times (RT) suggested by Ganel and Goshen-Gottstein (2004) might tell us something about discriminability in quantity. But as a relative variable, manipulation of discriminability needs some kind of direct assessment in quality. In our experiments, we employed an operational way to manipulate the discriminability of facial identity and facial expression besides selective RTs measurement. For facial identity, we obtained low and high discriminability by matching similar models and unlike models. Specifically, we selected similar models mainly by face shape. Both long and both round faces were matched in similar face pairs (similar face shape), and a long face matched with a round face in unlike face pairs (different face shape). Correspondingly, in expression recognition, we matched very strong happy with very strong angry to show high discriminability of expression. Particularly, we matched opened-mouth happy with closed-mouth angry in condition of high discriminability of expression and matched closed-mouth happy with closed-mouth angry in low discriminability of expression.

In addition to matches, we also assessed the selective reaction times of each matched face pair. Small mean RT was confirmed under high discriminability condition, and large mean RT was selected as low discriminability. Based on these manipulations, we finally selected four groups of facial photos as: high identity and low expression, low identity and high expression, low identity and low expression, and high identity and high expression. Following Experiment 1–4 were conducted with these four groups of facial photos respectively.

**FIGURE 1:**
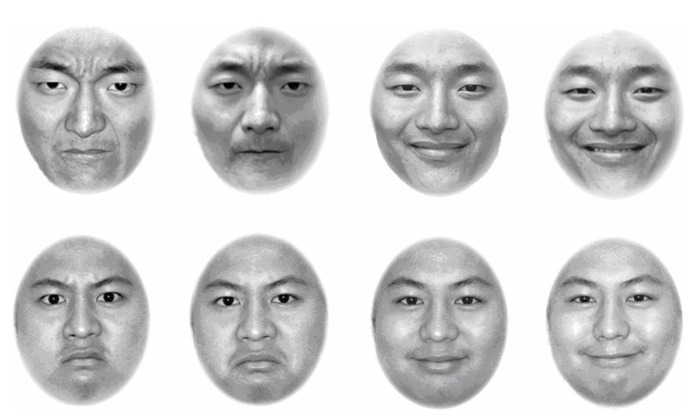
**Eight face photos used in Experiment 1.** The top row of the four photos comes from Model A, and the bottom row of the four photos comes from Model B. In both the top and bottom rows, the first two photos show angry expressions, with a small difference in emotional intensity between them, and the last two photos show happy expressions, also with a small difference in emotional intensity between them. The discriminability of expression was low, with a RT of 538 ms. and the discriminability of identity was high, with a RT of 475 ms.

As shown in **Figure 1**, the stimuli used in Experiment 1 were eight face photos of two young male models, named model A and model B. Each model was photographed with a happy expression and with an angry expression. To discourage participants’ use of picture-based strategies (i.e., memorizing selected pictures rather than classifying different identities or expressions), we used two versions of each facial expression of each model. Thus, the stimuli consisted of eight monochrome photos, four of Model A (two angry, two happy) and four of Model B (two angry, two happy). To prevent hairstyle-based strategies (i.e., using hairstyles to judge identities), we removed hair and hair-line contours from the photos using Adobe Photoshop (Version 7). All photos were of equal size: 465 pixels long and 350 pixels wide with an oval face area of 295 pixels. The color of the background was white. The contrast between the faces and the background was kept constant.

Photos used in Experiment 1 are expected to be high discriminability in identity and low discriminability in expression. Operationally, we matched a long face with a round face for high discriminability in identity, and a closed-mouth happy with a closed-mouth angry for low discriminability in expression at the same time. Thus, long face of model A was matched with round face of model B and closed-mouth happy was matched with closed-mouth angry (see **Figure 1**). After matching, we assessed selective RTs to confirm that small mean selective RT was made for high discriminability and large selective RTs were obtained for low discriminability. Twenty-four participants contributed to data of discriminability assessment (see **Table 1**).

**Table 1:** Mean reaction times (in milliseconds), standard errors (in parentheses), and percentages of error of performance in both identity- and expression-judgment tasks during discriminability assessment and interference measurements of Experiment 1.

	Block type							
Task	Discriminability		Baseline		Filtering		Interference^*^	
	RTs	% error	RTs	% error	RTs	% error	RTs	% error
Expression	538(21)	3.8	512(23)	2.7	558(20)	3.5	46	0.5
Identity	488(14)	2.0	473(14)	1.7	479(16)	1.8	6	0.1

* Garner interference = filtering RTs - baseline RTs.

The results showed that the discriminability of identity was high, with a short mean RT (*M* = 488, SE = 14), while the discriminability of expression was low, with a long mean RT (*M* = 538, SE = 21), yielding a 50-ms difference. The results of a *t*-test indicated that the discriminability of expression was significantly lower than that of identity, *t*(22) = 2.55, *p* < 0.05. Error rates of identity was lower than expression, *t*(22) = 2.50, *p* < 0.05. Small or large RT can be roughly judged based on previous study which used the same procedure and made a manipulation to discriminability (Ganel and Goshen-Gottstein, 2004). The results of RT assessment were in line with face matching in that long and round face match actually rendered a high identity discriminability while both closed mouth match led to a low expression discriminability. According to the first prediction, in such a combined condition, identity would interfere with expression recognition but expression would not interfere with identity recognition.

#### Design

In Experiment 1, block (baseline, filtering) was designed within participants and task (identity judgment, expression judgment) was conducted between subjects. Two blocks of Garner task, baseline block and orthogonal block (filtering block), were arranged (Garner, 1974). In the baseline block, participants judged one dimension (identity or expression), while the other dimension was held at a constant value (either Model A or Model B was presented). Thus, baseline block included two similar parts. For example, in identity recognition task, baseline block included two parts: identity recognition on happy face photos and identity recognition on angry face photos. In the filtering (orthogonal) block, participants again judged one dimension, but the stimuli varied along the other dimension (both Model A and Model B were presented). As a result, each part of the baseline block consisted of four face photos, and the filtering block consisted of eight photos. Within each block, each photo was presented 24 times in random order, resulting in a total of 96 presentations for each part of baseline block and 192 presentations for filtering block.

In our design, the task (identity, expression) was conducted between subjects by considering possible interactions between task responses as previous studies revealed (Kaufmann, 2002). For example, participant who just finished facial expression judgment was asked to judge facial identity with the same photos, the tendency of selectively attending to expression might continue to exist.

#### Procedure

The entire experiment consisted of two sessions: pre-experiment practice and Garner effect measurement. As introduced above, 24 participants took part in this experiment, half of them were assigned to the identity judgment task and the other half completed the expression judgment task. For participants either assigned to identity judgment task or assigned to expression judgment task, a practice session was arranged to acquaint them with the procedure and stimuli. All participants were unacquainted with the models before experiment. In practice session, participants were instructed to first learn some photos and then finish a recognition test. Following the instruction, eight photos were shown in pairs (two happy or two angry) with word captions, and each pair of photos was presented side by side on the computer screen for 5 s. Participants were asked to try to learn and discriminate these faces during presentation. After all eight photos were presented a recognition task followed. In this task, photos appeared in the centre of a computer screen for 2500 ms one by one or disappeared after a response. Each of the eight photos appeared two times, and feedback displayed after each incorrect response. Participants were instructed to judge identity in identity judgment task or expression in expression judgment task as quickly and accurately as possible by pressing an assigned key. Participants who were able to judge all faces correctly then attended the Garner effect measurement session.

In Garner effect measurement session, Garner task was performed with a baseline block (include two parts) and a filtering block (orthogonal block). Two parts of baseline block and filtering block were arranged in a random order. Each base line block began with eight practice trials (two random repetitions of each of four stimuli), and each filtering block began with 16 practice trials (two random repetitions of each of eight stimuli). Participants were given a shorter break between blocks.

Each Garner task trial began with a sign of cross for 500 ms, immediately followed by a blank white screen for 500 ms before face photos appeared on the screen. Each face photo was displayed at the center of the screen for 2500 ms at a resolution of 800 by 600 pixels on 17-in. screen. The visual angle subtended by face photo was about 10°. Face photo disappeared after a press or after 2500 ms. Participants pressed the right-hand key or the left-hand key respectively to judge identity (Model A or Model B in identity judgment task) or expression (happy or angry in expression judgment task). Arrangement of the right-hand key and the left-hand key was balanced between participants.

### RESULTS AND DISCUSSION

**Table 1** is a summary of descriptive statistics of mean RTs and percentages of error of participants’ performances. It addresses two specific questions: (1) Was there Garner interference from identity to expression recognition under the condition of low discriminability in expression and high discriminability in identity? (2) Was there a Garner inference from expression to identity recognition under the condition of low discriminability in expression and high discriminability in identity?

First, as shown in **Table 1**, for the expression judgment task, the mean RT in the baseline block (*M* = 512, SE = 23, see **Table 1**) was shorter than that in the filtering block (*M* = 558, SE = 20), leading to the 46-ms difference. Mean reaction times were subjected to a repeated-measures analysis of variance (RM-ANOVA). The analysis of variance indicated that the difference was significant, *F*(1,11) = 19.10, *p* < 0.01, η^2^ = 0.64. Despite the relatively small number of errors, a subsidiary RM-ANOVA was conducted on error rates. No speed-accuracy tradeoff found, *F*(1,11) = 1.98, *p >* 0.05, η^2^ = 0.15. Given identities did not vary in the baseline block but did vary in the filtering block, the difference in mean RT between these two types of blocks was due to the Garner interference of varying identity (Model A or B) on expression judgment. Thus, it is concluded that Garner interference from identity to expression was present.

Second, for the identity judgment task, the mean RT in the baseline block (*M* = 473, SE = 14) was similar to that in the filtering block (*M* = 479, SE = 16), leading to only a 6-ms difference. The analysis of variance (RM-ANAVOA) indicated that this difference was not significant, *F*(1,11) = 0.489, *p* > 0.05, η^2^ = 0.04. Despite the relatively small number of errors, a subsidiary RM-ANOVA was conducted on error rates. No speed-accuracy tradeoff was found, *F*(1,11) = 0.19, *p >* 0.05, η^2^ = 0.02. Since expressions did not vary in the baseline block but did vary in the filtering block, the difference in mean RTs between these two types of blocks was due to the Garner interference of varying expression (happy or angry) on identity judgment. Thus, it is concluded that Garner interference from expression to identity was not observed.

Based on the above results, we conclude that, using stimuli with low discriminability in expression and high discriminability in identity, identity interfere with but is not affected by expression recognition. The results confirm our first prediction and replicate the typical findings regarding the asymmetric relation hypothesis (Schweinberger and Soukup, 1998; Schweinberger et al., 1999). A follow-up question is: if the combination pattern of discriminability was reversed, would the pattern of interference be reversed too? To test this question, we conducted Experiment 2.

## EXPERIMENT 2

This experiment was designed to test the second prediction of the mediating discriminability hypothesis: when the discriminability of facial expression is high and the discriminability of facial identity is low, expression recognition will interfere with identity recognition but identity will not interfere with expression recognition.

Previous researchers had demonstrated the interference from identity to expression or to each other, but they failed to reveal the unidirectional interference from expression to identity (Schweinberger and Soukup, 1998; Schweinberger et al., 1999; Ganel and Goshen-Gottstein, 2004; Kaufmann and Schweinberger, 2004). It seemed that facial expression recognition always referenced to identity. As far as human perception is concerned, hundred-percent asymmetric interference might attenuate its flexibility in adapting environment (Calder and Young, 2005). Signals on facial expression are sometime top priority for our survival. Therefore, we can reasonably infer that facial expression would be processed independent of facial identity under certain condition. In this sense, Experiment 2 is a key part of our hypothesis.

This experiment used the same design and procedure as Experiment 1, except that the stimuli had high discriminability in expression and low discriminability in identity.

### METHOD

#### Participants

Forty-eight undergraduates with normal or corrected-to-normal vision participated in the experiment, a sample different from the one used in Experiment 1. Half participated in discriminability assessment and the other half contributed to Garner effect measurements. Among the participants for discriminability assessment or the participants for Garner tasks, half assigned to the identity judgment tasks and the other half completed the expression judgment tasks. We used the results of discriminability assessment to show discriminability levels of selected facial photos, and used the results of Garner effect measurements to test the second prediction.

#### Stimuli

**FIGURE 2:**
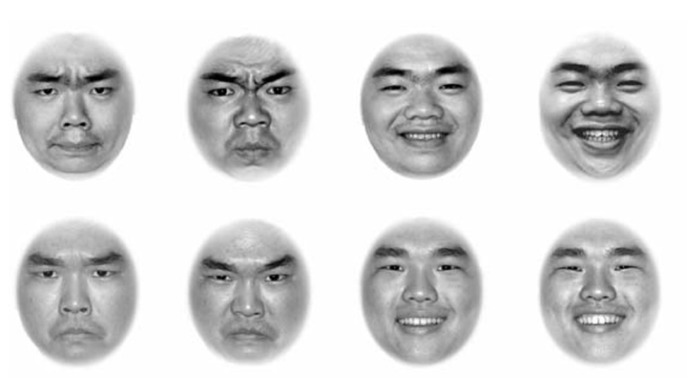
**Eight face photos used in Experiment 2.** The top row of the four photos comes from Model C, and the bottom row of the four photos comes from Model D. In both the top and bottom rows, the first two photos show angry expressions, with a small difference in emotional intensity between them, and the last two photos show happy expressions, also with a small difference in emotional intensity between them. The discriminability of expression was high, with a RT of 440 ms. and the discriminability of identity was low, with a RT of 519 ms.

As shown in **Figure 2**, stimuli were eight face photos, four of Model C (two angry, two happy), and four of Model D (two angry, two happy). These photos were designed to be comparable with those used in Experiment 1 but the discriminability level of identity and expression was reversed. We selected two round faces for identity, and matched closed-mouth angry with opened-mouth happy for expression. Twenty-four participants contributed to the data of discriminability assessment.

The results of discriminability assessment showed that the discriminability of identity was relatively low, with a long mean RT (*M* = 519, SE = 13), while that of expression was relatively high, with a short mean RT (*M* = 469, SE = 20). The results of a *t*-test indicated that the discriminability of expression was higher than that of identity, *t*(22) = 2.03, *p* < 0.05. Error rates showed no difference, *t*(22) = 0.95, *p* > 0.05. Manipulations of matching were confirmed in that identity discriminability was low while expression discriminability was high. According to the second prediction, in such a condition, expression would interfere with identity recognition but identity would not interfere with expression recognition.

### RESULTS AND DISCUSSION

**Table 2** is a summary of descriptive statistics of mean RT and percentage of errors of participants’ performances, suggesting two major findings of the experiment.

**Table 2:** Mean reaction times (in milliseconds), standard errors (in parentheses), and percentages of error of performance in both identity- and expression-judgment tasks during discriminability assessment and interference measurements of Experiment 2.

	Block type							
Task	Discriminability		Baseline		Filtering		Interference^*^	
	RTs	% error	RTs	% error	RTs	% error	RTs	% error
Expression	469(20)	2.3	453(17)	2.0	462(16)	2.1	9	0.1
Identity	519(13)	3.2	517(21)	2.0	538(26)	2.3	21	0.3

* Garner interference = filtering RTs - baseline RTs.

First, for the expression judgment task, the mean RT in the baseline block (*M* = 453, SE = 17) was similar to that in the filtering block (*M* = 462, SE = 16), leading to only a 9-ms difference. Mean reaction times were subjected to a RM-ANOVA. The analysis of variance indicated that this difference was not significant, *F*(1,11) = 1.93, *p* > 0.05, η^2^ = 0.15. Despite the relatively small number of errors, a subsidiary RM-ANOVA was conducted on error rates. No speed-accuracy tradeoff was found, *F*(1,11) = 0.20, *p* > 0.05, η^2^ = 0.02. Thus, it is concluded that the Garner interference from identity to expression was not present.

Second, for the identity judgment task, the mean RT in the baseline block (*M* = 517, SE = 21) was shorter than that in the filtering block (*M* = 538, SE = 26), leading to the 21-ms difference. Mean reaction times were subjected to a RM-ANOVA. The analysis of variance indicated that this difference was not significant, *F*(1,11) = 7.76, *p* < 0.05, η^2^ = 0.41. Despite the relatively small number of errors, a subsidiary RM-ANOVA was conducted on error rates. No speed-accuracy tradeoff was found, *F*(1,11) = 0.95, *p* > 0.05, η^2^ = 0.08. It is thus concluded that the Garner interference from expression to identity was present.

The results of Experiment 2 validate our second prediction, that is, expression with high discriminability interferes with but is not affected by identity with low discriminability. Reversing the pattern of discriminability selected in Experiment 1 led to a reversed pattern of interference in Experiment 2. This is the first report that interference emerged just from expression to identity.

Previous studies mainly operated the discriminability of identity but failed to manipulate identity and expression simultaneously. To some extent, how facial expression is recognized depends on the discriminability of expression. According to evolution theory, some kind of emergency makes facial expression recognition a top priority. It suggests that facial expression might be processed independent of identity.

The findings of this experiment can be considered as evidence supporting the hypothesis that low discriminability causes interference. However, another possible interpretation is that relatively lower discriminability between two dimensions (e.g., lower identity discriminability as compared with higher expression discriminability) but not relatively low discriminability within dimension caused interference. Thus, it is necessary to examine whether low discriminability in both identity and expression would cause interference to each other. To answer this question, we then conducted Experiment 3.

## EXPERIMENT 3

Experiment 3 was designed to test the third prediction of the mediating discriminability hypothesis: when the discriminability of facial expression and facial identity are both low, expression interferes with identity recognition and identity interferes with expression recognition. The design and procedure was identical to Experiment 1, except that all face photos used in the experiment had relatively low discriminability in expression and low discriminability in identity.

### METHOD

#### Participants

Forty-eight undergraduates with normal or corrected-to-normal vision were recruited, a sample different from the previous two experiments. Among them, 24 took part in discriminability assessment (12 for the identity judgment task and 12 for the expression judgment task), and the other 24 participated in the Garner effect measurements (12 for the identity judgment task and 12 for the expression judgment task).

#### Stimuli

**FIGURE 3:**
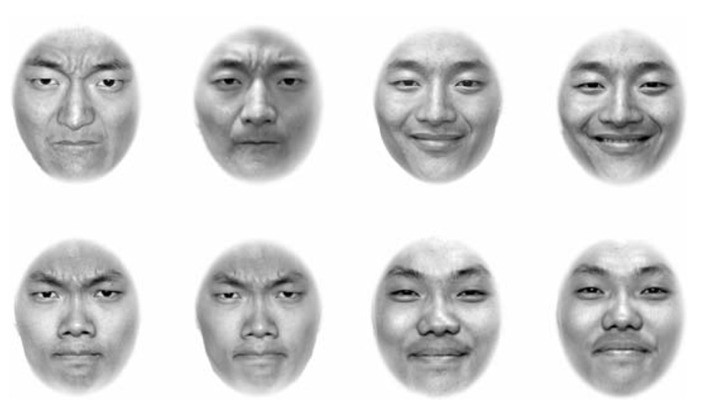
**Eight face photos used in Experiment 3.** The top row of the four photos comes from Model A, and the bottom row of the four photos comes from Model E. In both the top and bottom rows, the first two photos show angry expressions, with a small difference in emotional intensity between them, and the last two photos show happy expressions, also with a small difference in emotional intensity between them. The discriminabilities of expression and identity were both low, with RT of 530 ms and 550 ms.

As shown in **Figure 3**, the stimuli used were eight face photos, four of Model A (two angry, two happy) and four of Model E (two angry, two happy). These photos were designed to have low discriminability in expression and low discriminability in identity. For identity, two long faces were matched, and for expression, closed-mouth happy matched with closed-mouth angry. Twenty-four participants contributed to the data of discriminability.

The results of discriminability assessment showed that the discriminability of identity was low, indicated by a long mean RT (*M* = 561, SE = 22), and the discriminability of expression was also low, indicated by a long mean RT (*M* = 546, SE = 23). The results of a *t*-test indicated that the discriminability of expression was similar to that of identity, *t*(22) = -0.49, *p >* 0.05. Error rates showed no difference, *t*(22) = –0.58, *p >* 0*.*05. Matching manipulations were confirmed in that either identity or expression had a relatively low discriminability. According to our third prediction, in such a condition, expression and identity would interfere with each other in the two directions.

### RESULTS AND DISCUSSION

**Table 3** is a summary of descriptive statistics of mean RT and percentage of errors of participants’ performances. It presents two findings of the experiment.

**Table 3:** Mean reaction times (in milliseconds), standard errors (in parentheses), and percentages of error of performance in both identity- and expression-judgment tasks during discriminability assessment and interference measurements of Experiment 3.

	Block type							
Task	Discriminability		Baseline		Filtering		Interference^*^	
	RTs	% error	RTs	% error	RTs	% error	RTs	% error
Expression	546(23)	3.1	529(14)	1.9	545(15)	1.8	16	–0.1
Identity	561(22)	2.5	579(16)	1.8	620(21)	2.4	41	0.6

* Garner interference = filtering RTs - baseline RTs.

First, for the expression judgment task, the mean RT in the baseline block (*M* = 529, SE = 14) was similar to that in the filtering block (*M* = 545, SE = 15), leading to the 16-ms difference. Mean reaction times were subjected to a RM-ANOVA. The analysis of variance indicated that this difference was significant, *F*(1,11) = 7.85, *p* < 0.05, η^2^ = 0.42. Despite the relatively small number of errors, a subsidiary RM-ANOVA was conducted on error rates. No speed-accuracy tradeoff was found, *F*(1,11) = 0.10, *p* > 0.05, η^2^ = 0.01. Thus, it is concluded that there was significant Garner interference from expression to identity.

Second, for the identity judgment task, the mean RT in the baseline block (*M* = 579, SE = 16) was shorter than that in the filtering block (*M* = 620, SE = 21), leading to the 41-ms difference. Mean reaction times were subjected to a RM-ANOVA. The analysis of variance indicated that this difference was significant, *F*(1,11) = 13.90, *p* < 0.01,η^2^ = 0.56. Despite the relatively small number of errors, a subsidiary RM-ANOVA was conducted on error rates. No speed-accuracy tradeoff was found, *F*(1,11) = 3.47, *p* > 0.05,η^2^ = 0.24. Thus, it is concluded that there was also significant Garner interference from identity to expression.

The results of Experiment 3 support our third predictions that, with low discriminability of identity and expression, expression and identity interfere with each other in the two directions. These results suggest that the relationship between expression and identity recognition is not asymmetric as previous studies suggested (Schweinberger and Soukup, 1998; Schweinberger et al., 1999), and reducing discriminability of identity actually cause interference from expression to identity (Ganel and Goshen-Gottstein, 2004). The similar results were also revealed in recognition of emotion and gaze (Graham and LaBar, 2007). More importantly, the results of Experiment 3 indicate that it is not lower discriminability between dimensions (e.g., expression is lower than identity) but low discriminability within dimension that caused Garner interference.

Results from Experiments 1–3 indicate that low discriminability cause interference. It suggests that low discriminability of one dimension demands a more elaborate process and then makes a reference to the other dimension. Because this process is highly demanding, elaborate, and difficult, it will be easily interfered by other irrelevant facial cues, regardless of expression (see Task Expression in **Table 3**) or identity (see Task Identity in **Table 3**) judgment tasks. This explanation leads to another question: if both facial identity and facial expression are highly discriminable, would Garner interference still exist with either one? To further answer this question, we conducted Experiment 4.

## EXPERIMENT 4

Experiment 4 aimed to test the fourth and the last prediction of the mediating discriminability hypothesis: facial identity and expression recognition are independent of each other when both have high levels of discriminability.

### METHOD

#### Participants

Forty-eight undergraduates with normal or corrected-to-normal vision were recruited, a sample different from the previous three experiments. Among them, 24 took part in the discriminability assessments, and the remaining 24 participated in the Garner effect measurements.

#### Stimuli

**FIGURE 4:**
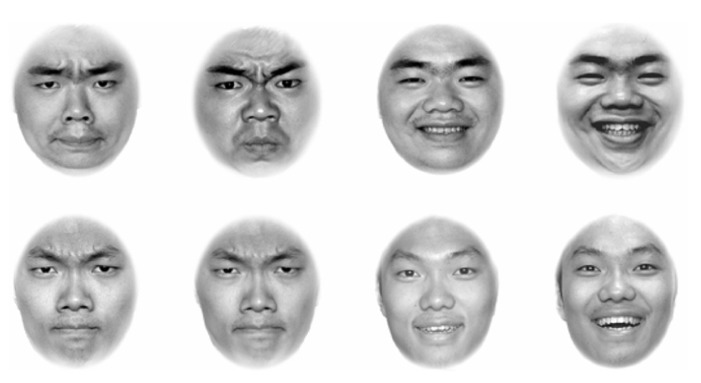
**Eight face photos used in Experiment 4.** The top row of the four photos comes from Model C, and the bottom row of the four photos comes from Model E. In both the top and bottom rows, the first two photos show angry expressions, with a small difference in emotional intensity between them, and the last two photos show happy expressions, also with a small difference in emotional intensity between them. The discriminabilities of expressions were both high, with RT of 467 ms and 480 ms.

As shown in **Figure 4**, the stimuli used were eight face photos, four of Model C (two angry, two happy) and four of Model E (two angry, two happy). These photos were designed to have high discriminability in expression and high discriminability in identity. We matched round face with round face for identity, and matched closed-mouth angry with opened-mouth happy for expression. Twenty-four participants contributed to the data of discriminability.

The results of the discriminability assessment showed that the discriminability of identity was high, with a short mean RT (*M* = 480, SE = 13), and that of expression was also high, with a short mean RT (*M* = 467, SE = 14). The results of a *t*-test further indicated that the discriminability of expression was similar to that of identity, *t*(22) = 0.68, *p* > 0.05. Error rates showed no difference, *t*(22) = 0.01, *p* > 0.05. Matching manipulations were confirmed in that either identity or expression had a high discriminability. According to the fourth prediction, in such a condition, expression would not interfere with identity recognition and identity would not interfere with expression recognition.

### RESULTS AND DISCUSSION

**Table 4** is a summary of descriptive statistics of mean RT and percentage of errors of participants’ performances. It presents two findings of the experiment.

**Table 4:** Mean reaction times (in milliseconds), standard errors (in parentheses), and percentages of error of performance in both identity- and expression-judgment tasks during discriminability assessment and interference measurements of Experiment 4.

	Block type							
Task	Discriminability		Baseline		Filtering		Interference^*^	
	RTs	% error	RTs	% error	RTs	% error	RTs	% error
Expression	467(14)	1.9	498(23)	2.7	499(27)	3.0	1	0.3
Identity	480(13)	1.9	495(16)	1.2	502(18)	1.0	7	–0.2

* Garner interference = filtering RTs - baseline RTs.

First, for the expression judgment task, the mean RT of the baseline block (*M* = 498, SE = 23) was similar to that in the filtering block (*M* = 499, SE = 27), with only a 1-ms difference. Mean reaction times were subjected to a RM-ANOVA. The analysis of variance indicated that this difference was not significant, *F*(1,11) = 0.07, *p* > 0.05,η^2^ = 0.01. Despite the relatively small number of errors, a subsidiary RM-ANOVA was conducted on error rates. No speed-accuracy tradeoff was found, *F*(1,11) = 0.42, *p >* 0.05,η^2^ = 0.04. The result showed that no Garner interference existed from identity to expression.

Second, for the identity judgment task, the mean RT in the baseline block (*M* = 495, SE = 16) was close to that in the filtering block (*M* = 502, SE = 18), with only a 7-ms difference. Mean reaction times were subjected to a RM-ANOVA. The analysis of variance indicated that this difference was not significant, *F*(1,11) = 0.50, *p* > 0.05,η^2^ = 0.04. Despite the relatively small number of errors, a subsidiary RM-ANOVA was conducted on error rates. No speed-accuracy tradeoff was found, *F*(1,11) = 0.30, *p >* 0.05,η^2^ = 0.03. The result supported that no Garner interference existed from expression to identity.

The results of Experiment 4 support the fourth prediction. High discriminability leads to independent processing. This experiment, together with the three other experiments, demonstrated four possible interference patterns (i.e., interference from identity to expression, from expression to identity, in both directions, and in either directions), which match discriminability patterns. These results indicate that low discriminability is truly responsible for the interference effects in recognition of identity and expression.

**FIGURE 5:**
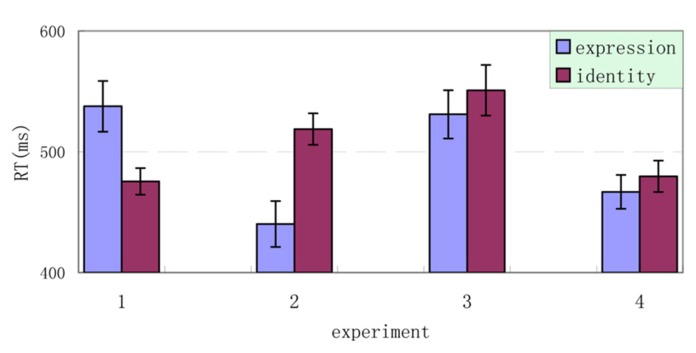
**Four combinations of discriminability levels of facial identity and expression used in Experiments 1 through 4: (1) low discriminability in expression with high discriminability in identity (Experiment 1), (2) high discriminability in expression with low discriminability in identity (Experiment 2), (3) low discriminability in expression with low discriminability identity (Experiments 3), and (4) high discriminability in expression with high discriminability in identity (Experiment 4).** The level of discriminability was measured with reaction time, where approximately 500 ms was used in the study as a dividing line to determine whether discriminability was high or low (see Results and Discussion of Experiment 4 for details).

When interpreting the findings, two possible issues exist. The first issue is how relatively low or high discriminability was defined in Experiments 1–4. In quality, we manipulated the discriminability of identity and expression by matching face shape in identity and mouth performing in expression. Face shape is widely used to describe facial identity in our daily life. Opened or closed-mouth used in our operation normally show us the intensity of expression. Selective RTs further confirmed in quantity in that: (1) difference between high and low discriminability in each experiment was significant; (2) results of Experiment 1 replicated the frequently findings of asymmetric interference, and the discriminability assessments in this experiment were statistically confirmed as high for identity and low for expression; (3) analysis among discriminabilities of four experiments was made to show the relative level in high or low, see illustration in **Figure 5**. The post hoc analysis using LSD multiple comparison indicated that discriminations of expression selected in Experiments 1 and 3 were significantly higher than that selected in Experiments 2 and 4 (α = 0.05), and the discriminability of identity selected in Experiments 2 and 3 were substantially but not significantly higher than that selected in Experiments 1 and 4 (α = 0.10). These results provided evidence that the discriminability of expression in Experiments 1 and 3, and discriminability of identity in Experiments 2 and 3 were relatively high. Considering Experiment 1 as frequently demonstrated findings, we can make a judgment of high or low discriminabilities in Experiments 2–4. These results indicate that the manipulations of face shape and mouth performing are valid in selecting high or low discriminability.

The second issue is whether a happy expression would be a confounding factor influencing unfamiliar identity recognition, as suggested in several studies on familiar faces (e.g., Baudouin et al., 2000). To examine this issue, mean reaction times in the filtering block in four experiments were subjected to a RM-ANOVA with discriminability as the between-subject variable and categories of expression as the within-subject variable. The results showed that main effect of discriminability was significant, *F*(3,44) = 5.13, *p* < 0.05,η^2^ = 0.26, the main effect of expression was not significant, *F*(1,44) = 1.22, *p* > 0.05,η^2^ = 0.27, and the interaction effect was also not significant, *F*(3,44) = 0.36, *p* > 0.05,η^2^ = 0.02. These results showed that neither happy nor angry expressions affected unfamiliar facial identity recognition.

Base on the results of above four experiments, the hypothesis of mediating discriminability was well supported. Low discriminability either of identity or expression led to significant Garner interference. That means Garner interference does not necessarily relate to the interdependent relationship between facial identity and facial expression.

## GENERAL DISCUSSION

Based on previous studies, we demonstrated that discriminability of identity and expression mediates Garner interference in recognition of facial identity and expression. The results suggest a notable issue in Garner task: when we apply Garner paradigm to complex stimuli, factors like discriminability might be an important mediating factor. Our findings supported that Garner interference is not necessarily related to interdependent processing in recognition of facial identity and expression. Furthermore, discriminability as a mediating factor should be carefully controlled in future research in facial recognition field. Among our studies, there arouse several interesting issues that are worth more discussion and further exploring.

Fist, Garner task provides a pretty way to investigate relations between face properties. However, a limited number of stimuli used in base line and orthogonal line might attenuate its potential power. Increasing stimuli and controlling discriminability, it is the choice of be in a dilemma all the time. More repetitions with different face photos might help solve the dilemma.

Second, why is it easy to reveal interference from identity to expression? Inherently, we like to think that facial expression recognition is as easy as identity recognition, but that is not always evidence in facial recognition experiments. Firstly, facial expressions, unlike identity, are usually hard to recognize by static photos (Humphreys et al., 1993; Wang and Fu, 2007). We might use information of facial action and pose to recognize a facial expression in daily life, but such information is hard to obtain from a static face photo. The difference mostly contributes to the difference between identity and expression, and also accounts for the relatively low discriminability of expression in static facial photos. A similar notion was proposed in recognition of sex and emotion (Atkinson et al., 2005). Secondly, expression is variable while identity is invariable (Haxby et al., 2000), that make it easy to neglect intensity of expression. Among existing studies, intensity of facial expression was scarcely controlled comparing with the considering of similarity in facial identity (Schweinberger and Soukup, 1998; Schweinberger et al., 1999; Ganel and Goshen-Gottstein, 2004; Kaufmann and Schweinberger, 2004). The current study successfully increased the discriminability of expression by matching opened-mouth happy with closed-mouth angry, and demonstrated that expression recognition can be independent of identity.

Third, discriminability is essentially related to interference but not unique determinative factor. Familiarity is another complicated factor in facial recognition (see Calder and Young, 2005 for a review). The current work focused on unfamiliar facial recognition but practice before Garner task was used to acquaint participants with these facial identity and expression, which may have led to a kind of familiarity. Recognition test before experiments help control familiarity but not eliminate the possible influence of familiarity. Furthermore, existing facial recognition studies explored the role of familiarity in Garner interference (Ganel and Goshen-Gottstein, 2004; Kaufmann and Schweinberger, 2004), but few examined the interaction between discriminability and familiarity.

Fourth, index of discriminability needs more examination. In current work, we successfully manipulated the discriminability by combining qualitative and quantitative methods. Selective RT, as a quantitative scale, was valid to measure discriminability under certain condition (Ganel and Goshen-Gottstein, 2004; Graham and LaBar, 2007), but not adequate to be an index. Future work might focus on looking for a possible neuropsychological index to discriminability.

Finally, what is the possible mechanism underlying the Garner interference in facial recognition? Does the mediating discriminability hypothesis apply to non-face stimuli? Our experiments show that low discriminability causes Garner interference in facial recognition. But how does the low discriminability lead to interference? In terms of our definition, low discriminability means a difficult discrimination task, which suggests that limited information is available. Thus, it is reasonable to infer that Garner interference derives from an extra reference to irrelevant dimensional information. In fact, similar results were also found in non-face stimulus recognition. With compound stimuli, Pomerantz found that Garner interference in local discrimination was larger than in whole discrimination (Pomerantz, 1983). It is reasonable to assume that the discriminability of local discrimination is lower than that of whole discrimination.

## Conflict of Interest Statement

The authors declare that the research was conducted in the absence of any commercial or financial relationships that could be construed as a potential conflict of interest.
